# A Case Report of Pediatric Ovarian Torsion: The Importance of Diagnostic Laparoscopy

**DOI:** 10.5811/cpcem.2020.12.50319

**Published:** 2021-01-26

**Authors:** Alana Corre°, Shebani Dandekar°, Christopher Lau, Leonard Ranasinghe

**Affiliations:** * California Northstate University College of Medicine, Elk Grove, California; † Kaiser Permanente, Department of Emergency Medicine, Modesto, California

**Keywords:** Case report, pediatric, ovarian torsion

## Abstract

**Introduction:**

Pediatric ovarian torsion is a relatively rare occurrence with chances of significant morbidity and possible mortality if not treated emergently.

**Case Report:**

In this report, we review a case of pediatric ovarian torsion in a nine-year-old that was difficult to diagnose on initial presentation to the hospital due to various factors, which inevitably led to delayed resolution.

**Conclusion:**

We discuss the diagnosis of pediatric ovarian torsion including risk factors, symptoms, imaging modalities, and surgical diagnostics. To improve diagnosis and shorten time to treatment, this case supports the use of laparoscopy for diagnosis of ovarian torsion if indicated by clinical suspicion and supplemental imaging studies.

## INTRODUCTION

Pediatric ovarian torsion can be challenging to diagnose. We present a case of ovarian torsion (OT) in a nine-year-old premenarchal female with delayed diagnosis and treatment. We discuss the varied presentations, diagnostics, and imaging modalities used to assess pediatric patients with suspected OT. To improve OT diagnosis and time to treatment, our case supports the use of diagnostic laparoscopy if prompted by clinical suspicion and other studies.

## CASE PRESENTATION

A nine-year-old premenarchal female presented to the emergency department (ED) for intermittent, right lower quadrant abdominal pain that began two days prior. An abdominal and pelvic computed tomography (CT) with intravenous contrast showed a “7 × 7.5 [centimeter (cm)] right ovarian hypoenhancing structure with multiple peripheral and internal follicle-like structures highly suspicious for swollen ovary secondary to ovarian torsion.” A pelvic ultrasound completed after the CT showed right adnexal enlargement and “no evidence of ovarian torsion”; thus, no further work-up or consultations were pursued and the patient was discharged and instructed to follow up with her primary care physician (PCP) the next day.

At the appointment with her PCP, the patient was still in significant pain and was referred to the ED again, this time at a different hospital. At her second ED visit, the patient was nauseous without vomiting, and reported no fevers or chills. Her physical examination showed minimal diffuse abdominal tenderness worse in the right lower quadrant and moderate McBurney’s point tenderness with guarding. Labs revealed a white blood cell count within reference range, but the differential showed a high neutrophil count. A repeat ultrasound showed an “8.7 [× 1.3 × 6.1] cm heterogenous, mass-like structure containing small cystic foci and lack of blood flow” on her right ovary and a small amount of free fluid (Image).

A gynecologist was consulted and the patient was admitted and sent for an emergent diagnostic laparoscopy, during which OT was identified and corrected. The patient also required an ovarian cystectomy and partial right ovarian oophorectomy. Postoperatively, the patient recovered well and had no known complications or recurrence of disease as documented by her PCP and had not returned to the ED for any similar complaints in the following year. Since a partial oophorectomy was required, the long-term effects remain to be seen as the patient progresses into her childbearing years.

## DISCUSSION

The adnexa of the uterus, which consists of the ovaries, fallopian tubes, and supporting ligaments, can undergo torsion or twisting. In OT specifically, the ovary twists on its axis, causing occlusion of ovarian vessels, which can lead to ischemia and necrosis of the ovary, possibly causing infertility or death. Pediatric OT is rare with an incidence of 4.9 in 100,000 females ages one to 20 years-old.^1^ Although rare, pediatric OT is a clinical emergency requiring immediate diagnosis and treatment. However, the difficulty in diagnosing pediatric OT often leads to delayed treatment in youth. The risk factors for OT include the presence of an ovarian mass or lesion, having an enlarged ovary, being of reproductive age, pregnancy, ovulation induction, prior OT, tubal ligation, and polycystic ovarian syndrome. Up to 95% of patients found to have OT had an ovarian mass, making this the primary risk factor.^2^

Ovarian volume can also be measured to assess for risk of OT. Using the equation, length × width × height × 0.523 for measurement of the ellipsoid ovary, premenarchal girls two to 13 years of age have been found to have a mean ovarian volume of 0.7 to 4.2 cm^3^.^3^,^4^ Ovaries with a volume larger than 6.0 to 8.0 cm^3^ in premenarchal girls should raise suspicion for torsion.^5^ Our patient’s 7.0 × 7.5 cm cystic, swollen ovary revealed on her initial CT posed a major risk for OT and should have prompted immediate admission and treatment. The initial follow-up ultrasound detected a smaller 5.4 × 4.9 × 5.0 cm ovary. Although the ultrasound results showed no evidence of torsion, the ultrasound did confirm an enlarged right ovary with a volume of 69 cm^3^ placing the patient at increased risk for developing OT. Patients with normal ovaries (ovaries without a mass and not enlarged) are also at risk for developing torsion, particularly individuals in the pediatric population. In patients younger than 15 years-old, 50% of those who present with OT have normal ovaries.^6^ In addition to recognizing OT risk factors and the risk of OT in pediatric patients with normal ovaries, other indicators such as clinical symptoms should be assessed.

**CPC-EM Capsule**

What do we already know about this clinical entity?

Pediatric ovarian torsion is rare, difficult to diagnose, and can lead to significant morbidity and possible mortality if not identified and treated emergently.

What makes this presentation of disease reportable?

Initial diagnostic imaging was doubted and definitive treatment was delayed, highlighting the challenges in diagnosing this condition.

What is the major learning point?

To improve diagnosis and shorten time to treatment, laparoscopy is useful for diagnosis of ovarian torsion if indicated by clinical suspicion and supplemental imaging.

How might this improve emergency medicine practice?

Emergency clinicians will be better prepared to diagnose, stabilize, and obtain emergent surgical consultation on pediatric patients presenting with ovarian torsion.

Pediatric OT can have a multitude of presentations. In a literature review of pediatric OT, most patients presented with peripheral leukocytosis, lower quadrant abdominal pain without radiation, and vomiting.^7^ Another study found that vomiting, short duration of abdominal pain (less than six hours), and a high C-reactive protein level have a high predictive value when diagnosing OT.^8^ Clinical presentation of OT in the premenarchal population has been found to be different than the postmenarchal population. In a retrospective study including 41 premenarchal and over 200 postmenarchal patients, those who were premenarchal were more likely to present with restlessness, fever, and a palpable pelvic mass.^9^ The most common presentation among both groups was abdominal pain, although diffuse pain was more common in premenarchal while lower abdominal pain was more common in postmenarchal patients.

Even between premenarchal and postmenarchal populations, there are differences in triage and time to surgery for OT cases. For premenarchal patients (median age nine years old), the time from onset of symptoms to admission for OT was 28.5 hours on average, as compared to only seven hours for postmenarchal patients (median age 27 years-old).^9^ The time from admission to initiation of the surgical procedure in the operating room was 9.5 hours in the premenarchal group versus 4.6 hours in the postmenarchal group. Theories for these findings postulated in the study include that providers often do not consider OT as a possible diagnosis in the premenarchal population and providers have reservations about operating on premenarchal patients. However, the exact reasons for these differences were unknown due to limitations of the study.

In our case, the patient also presented at her second ED visit with diffuse abdominal tenderness increased in the right lower quadrant and nausea without vomiting. Our patient’s CT at the prior hospital was done about 26 hours before she was admitted to the operating room for a diagnostic laparoscopy, which thereby reinforces the difficulty of assessing for OT in premenarchal patients. In addition to symptoms, lab findings can also help to further elucidate the diagnosis and thus reduce time to surgery.

The patient had a normal leukocyte count for her age on initial presentation. However, her neutrophil to lymphocyte ratio (NLR) was high at 5.64 with neutrophils at 79% and lymphocytes at 14%. Many recent studies have found NLR to have diagnostic and prognostic value when assessing patients for OT. One study found that an NLR greater than 2.44 was the best predictor of OT when compared to other complete blood count values, such as red cell distribution width and platelet distribution width.^10^ This study also observed that NLR was higher in patients with ovarian necrosis as opposed to those without necrosis. The increased neutrophils and decreased lymphocytes are believed to be the result of ischemia in OT causing a cortisol stress response that leads to neutrophilia and lymphopenia.^10^ Based on these studies, our patient’s NLR could have been another factor pointing to her diagnosis of OT and the high likelihood of ovarian necrosis present.

Clinical suspicion for OT requires emergent work-up with imaging. Generally, ultrasound is considered the first-line tool for diagnosis. Ultrasound has been shown to be 92% sensitive and 96% specific in detecting adnexal torsion.^11^ Ultrasound is useful for diagnosis in that it can indicate size of the ovary, echogenicity, free pelvic fluid, ovarian lesions, and Doppler flow. A coiled or twisted vascular pedicle seen on ultrasound, called whirlpool sign, is highly specific for torsion.^12^ One caveat is that spontaneous ovarian detorsion may occur and can result in intermittent or self-resolving symptoms as well as negative imaging.

In a case study of spontaneous detorsion in a 10-year-old female, the patient had intermittent abdominal pain and a negative ultrasound and so she was subsequently discharged.^13^ In our case, the patient may have similarly had a negative ultrasound due to spontaneous ovarian detorsion. Computed tomography can also detect OT and, if it indicates OT, is considered an acceptable diagnostic tool when completed prior to ultrasound. Computed tomography can detect the size of the ovary, twisting of the pedicle, distended pedicle, ovarian lesion, edema, free fluid, and hemorrhagic infarction and necrosis. In our case, the patient had an initial CT that showed a cystic ovary of 7.0 × 7.5 cm with possible OT. However, the patient had a negative follow-up ultrasound with regard to organ perfusion.

Physicians might conduct a confirmatory ultrasound for diagnosis even after finding or suspecting OT on CT; yet CT has been found to be just as sensitive and specific for detecting torsion.^14^ One study of 20 cases of OT demonstrated that there was no significant difference in identifying torsion from CT compared to ultrasound images.^14^ This indicates that if torsion is found on CT, treatment should be initiated without completion of a confirmatory ultrasound. However, if the CT is negative for OT but OT is clinically suspected, ultrasound should be pursued. Despite the use of CT and ultrasound for diagnosis, a diagnostic laparoscopy is the ultimate standard for diagnosis and confirmation of OT via direct visualization.

According to the American College of Obstetricians and Gynecologists (ACOG) clinical guidelines for adolescents with adnexal torsion, including but not limited to ovarian torsion, “There are no clinical or imaging criteria sufficient to confirm the diagnosis of adnexal torsion. If adnexal torsion is suspected, timely intervention with diagnostic laparoscopy is indicated to preserve ovarian function and future fertility.”^15^ Additionally, ACOG continues by stating that adnexal torsion is in fact a diagnosis made by surgery, and that if a laparoscopy is negative for torsion in an adolescent or pediatric patient, this is acceptable given preserving ovarian function and fertility outweighs the risks of surgery.

## CONCLUSION

Pediatric ovarian torsion is an emergency that can present in multiple ways and thus can be difficult to diagnose. Clinical suspicion of OT should drive diagnostic laparoscopy. Imaging can be supplemental but should not be used to rule out pediatric OT in the setting of high clinical suspicion. With pediatric patients, there is more at stake given the risk of infertility. The benefit of early diagnostic laparoscopy should outweigh the risk of negative surgical findings.

## Figures and Tables

**Image:**
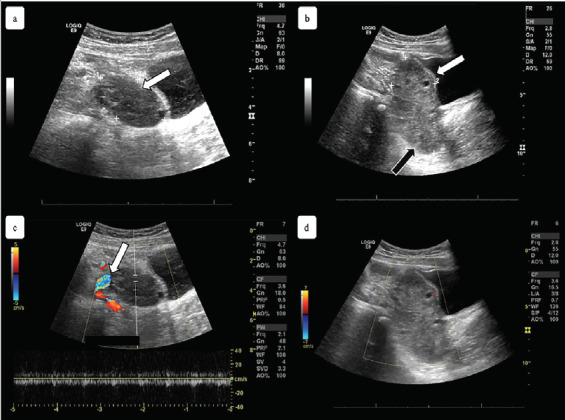
a) Sagittal view of the patient’s left ovary on transabdominal pelvic ultrasound, measuring 4.4 × 2.4 × 2.0 centimeters (cm), indicating a slightly enlarged but otherwise normal ovary (white arrow); b) sagittal view of the patient’s right ovary with measurements of 8.7 × 1.3 × 6.1 cm, demonstrating an abnormally enlarged ovary (white arrow) with a heterogenous mass (black arrow); c) sagittal view of the patient’s left ovary with normal color flow Doppler (white arrow); d) sagittal view of the patient’s right ovary demonstrating lack of blood flow on color flow Doppler.
